# Molecular diagnostics for perioperative microbial identification in periprosthetic joint infection: A scoping review and proposal of a diagnostic flow chart

**DOI:** 10.1002/jeo2.70263

**Published:** 2025-05-05

**Authors:** Pier Francesco Indelli, Trifon Totlis, Bruno Lovreković, Michael Engl, Bruno Violante, Pawel Skowronek, Guillaume Demey, Stefano Ghirardelli, Chiara Maci, Antonella Castagna, Laura Schianchi, Chiara Tassan Din, James Harty, Goksel Dikmen, Christian Schaller, Marko Ostojić

**Affiliations:** ^1^ European Society of Sports Traumatology, Knee Surgery Arthroscopy (ESSKA) – European Knee Associates (EKA) Board Luxembourg Luxembourg; ^2^ European Society of Sports Traumatology, Knee Surgery Arthroscopy (ESSKA) Basic Science Committee Luxembourg Luxembourg; ^3^ School of Medicine, Faculty of Health Sciences Aristotle University of Thessaloniki Thessaloniki Greece; ^4^ University Hospital “Merkur”, Zajčeva 19, Zagreb, Croatia and Faculty of Kinesiology Zagreb Croatia; ^5^ Adult Reconstruction and Joint Replacement Hospital for Special Surgery New York New York USA; ^6^ Paracelsus Medical University (PMU), Institute of Biomechanics Paracelsus Medical University Salzburg Austria; ^7^ Unit of Infectious and Tropical Diseases IRCCS San Raffaele Scientific Institute Milan Italy; ^8^ IRCCS Policlinico San Donato Milan Italy; ^9^ Department of Orthopedics and Traumatology, Faculty of Medicine, International Joint Center, Acibadem Maslak Hospital Acibadem University Istanbul Turkey; ^10^ Sports Traumatology Division, Traumatology Department "Draskoviceva" University Hospital "Sestre Milosrdnice" Zagreb Croatia; ^11^ Osteon Orthopedics and Sports Medicine Clinic Mostar Bosnia and Herzegovina

**Keywords:** molecular diagnostic, multiplex PCR, NGS, PCR, periprosthetic joint infection (PJI), THA, TKA

## Abstract

**Purpose:**

Periprosthetic joint infections (PJI) are among the most feared complications of joint reconstruction. Unfortunately, traditional cultures often fail to identify the aetiological agents of PJI. Molecular diagnostics can overcome the limitations of standard synovial fluid culture by utilising information from DNA/RNA samples to identify microbial species. The authors conducted a scoping review to evaluate the current state regarding the use of molecular diagnostics in the decision‐making process for the surgical treatment of PJI and to create a flowchart based on molecular diagnostics.

**Methods:**

A scoping review was conducted to provide an overview of the literature on molecular diagnostic techniques for detecting perioperative microbial infections in PJI. The population considered included patients undergoing total hip or knee arthroplasty or replacement, with a focus on molecular diagnostic methods within the perioperative period. The database search encompassed PubMed, Embase, Scopus and the Cochrane Library.

**Results:**

Seventy‐five articles were included after a preliminary review of 1315 records. Each article was assigned to one of four categories to fulfil the purpose of this review: (1) Polymerase chain reaction (PCR) related studies: *n* = 18; (2) Next‐Generation‐Sequencing (NGS) related studies: *n* = 40; (3) comparative studies, including systematic reviews and meta‐analyses, between different molecular diagnostic methodologies: *n* = 7; and (4) general reviews on nucleic acid‐based strategies to detect PJIs: *n* = 10.

**Conclusions:**

This review confirmed that molecular diagnostics are becoming extremely valuable tools in the decision‐making process for PJI treatment. Culture‐based techniques still represent the gold standard in PJI microorganism identification, but our review showed that standard culture, in 2025, could be integrated with newer nucleic acid‐based strategies.

**Level of Evidence:**

Level I.

**Abbreviations**
DAIR: debridement, antibiotics, implant retention
DAPRI: debridement, antibiotic pearls, retention of the implant
ESSKA: European Society of Sports Traumatology, Knee Surgery and Arthroscopy
mPCR: multiplex polymerase chain reaction
NGS: Next Generation Sequencing
PCC: population, concept, context
PCR: polymerase chain reaction
PJI: periprosthetic joint infections
THA: total hip arthroplasty
TJA: total joint arthroplasty
TKA: total knee arthroplasty

## INTRODUCTION

Periprosthetic joint infections (PJI) represent a catastrophic complication following total joint arthroplasty (TJA). The management of this complication has been the subject of international discussion: implant‐saving procedures have been recommended only in acute scenarios, while various surgical strategies have been proposed for revising the infected implant [48]. One‐stage, 1.5‐stage, and two‐stage revisions have been recommended based on several preoperative parameters, including host status, soft tissue appearance, microorganism identification and virulence, antibiotic sensitivity of microorganisms and finally, the extent of bone loss after implant removal. All these aspects require a multidisciplinary approach since orthopaedic surgeons often lack sufficient expertise to address all these aspects. A single‐stage revision represents the most desirable solution to this complication for surgeons and patients. This surgical approach has strict limitations: [31] microorganism identification, microorganism sensitivity to antibiotics, absence of generalised sepsis, healthy and sufficient soft tissue coverage, and absence of a sinus tract. Unfortunately, preoperative identification of the microorganism has been reported only in a limited percentage of PJI patients since culture yields negative results in up to 50% of PJIs [66]. While previous recommendations suggested that culture‐negative PJI is a contraindication for one‐stage exchange, recent studies have demonstrated favourable surgical outcomes in such cases. These findings suggest that patients without an identified infecting microorganism are not strictly limited to two‐stage revision.

The recent introduction of molecular diagnostic technologies has created a growing interest in adult reconstruction. DNA—and RNA‐based assays have recently been used in the strict perioperative period (including intraoperative) to identify the infecting microorganisms and their antibiotic resistance [20]. The most promising molecular techniques are broad‐range polymerase chain reaction (PCR) and Next‐Generation Sequencing (NGS). The latter allows for whole genome sequencing and rapid identification of antibiotic resistance by querying a database.

Few researchers [21, 57], including the current authors, have proposed PJI diagnostic protocols that utilise molecular diagnostic technologies on synovial fluid to identify microorganisms in the strict perioperative period and support the selection of the most appropriate surgical solution during PJI revision surgery.

Researchers from several European institutions, including two committees of the European Society of Sports Traumatology Knee and Arthroscopy (ESSKA)—the European Knee Associates (EKA) and the ESSKA Basic Science Committee—decided to conduct this scoping review to assess the current state of molecular diagnostics in the decision‐making process for the surgical treatment of periprosthetic joint infections. The authors aimed to review systematic reviews (SR), meta‐analyses (MA), general reviews (GR) and relevant articles published on this topic. Additionally, this study's secondary purpose was to propose a molecular‐diagnostic‐based diagnostic flow chart for PJI.

## MATERIALS AND METHODS

### Design

A scoping review was conducted to provide an overview of the current literature on molecular diagnostic techniques for detecting perioperative microbial infections in PJI. We adopted the Population, Concept, Context (PCC) framework to guide our search strategy. The population considered included patients with total knee arthroplasty (TKA), total hip arthroplasty (THA), total knee replacement (TKR) or total hip replacement (THR) and PJI; the concept was molecular diagnostic techniques; and the context of the perioperative period. This scoping review complies with the guidelines of the Joanna Briggs Institute (JBI) [1]. The Preferred Reporting Items for Systematic Reviews and Meta‐Analysis, an extension for Scoping Reviews (PRISMA‐ScR) [67] was applied to structure both the research reporting and the presentation of results. The leading institution's institutional review board (IRB) approved this scoping review.

### Eligibility criteria

The following eligibility criteria guided the study selection process: studies published in the last 5 years, including recent technological advances, available in English, and focusing on different molecular diagnostic techniques to detect pathogens in PJI. There were no restrictions on study design or country of origin. Studies were excluded if they examined diagnostic techniques that merely detected the presence of the pathogen without addressing its specific identification or if they did not concentrate on the perioperative period. Included articles reported on synovial fluid analysis only.

### Information sources

We conducted a thorough search for relevant evidence across the following electronic databases: PubMed, Embase, Scopus and the Cochrane Library. Additionally, we manually examined the reference lists of the studies included in our review to ensure comprehensive inclusion of pertinent research. This strategy also included grey literature sources, such as conference proceedings, dissertations and reports from relevant healthcare organisations and research institutes.

### Search strategy

The search strategy was developed using the PCC framework, as detailed below, and tailored for each electronic database. Articles focusing on the perioperative period were selected manually. The Boolean operator ‘AND’ was used to combine the PCC criteria, while ‘OR’ increased the sensitivity of the search query within each criterion. Medical Subject Headings (MeSH) terms were systematically integrated along with additional synonyms to enhance the precision and comprehensiveness of the search. The search queries were last updated on 15 September 15 2024. The search terms are included in Supporting Information: S1.

### Study selection process

Records retrieved from electronic databases were managed using EndNote 20 as reference management software, while additional studies identified through other searches were added manually. Duplicate entries from electronic databases were removed at the outset. During the screening phase, two authors independently assessed the titles and abstracts of studies using a double‐blind approach in the initial round. The first author (PFI) represents the European Society for Sports Traumatology, Knee Surgery, and Arthroscopy (ESSKA) Basic Science Committee, while the second (BV) represents the ESSKA—EKA Board. Discrepancies between reviewers were resolved through consensus discussions in subsequent rounds. Studies meeting the predefined eligibility criteria underwent a full‐text review to confirm their inclusion. A third author (SG) independently reviewed the final set of included studies to ensure consistency. The entire study selection process adhered to the PRISMA‐ScR guidelines [67].

### Data charting process

Data from the included articles were systematically organised using a predefined data charting form in Microsoft Excel, which captured details such as study design, primary focus, presence of a control group, and types of outcomes assessed. This structured approach ensured the consistent extraction and categorisation of relevant information from the primary studies, facilitating a comprehensive analysis.

### Data synthesis

To facilitate data synthesis, the articles were categorised into four main groups based on their topic and study design. The data were synthesised narratively.

## RESULTS

### Study selection

**Figure 1:**
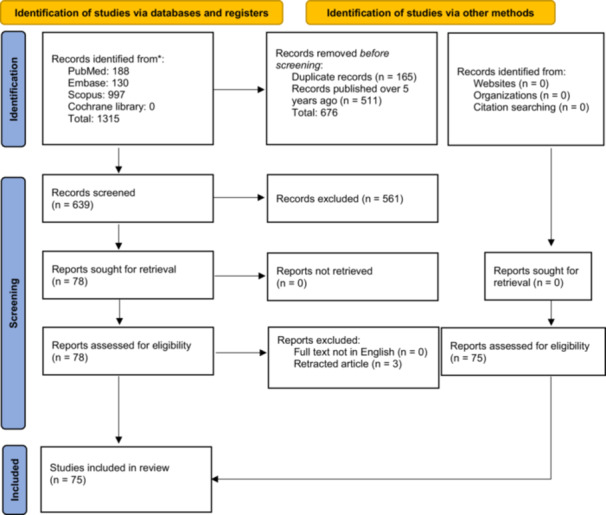
PRISMA flowchart.

**Figure 2:**
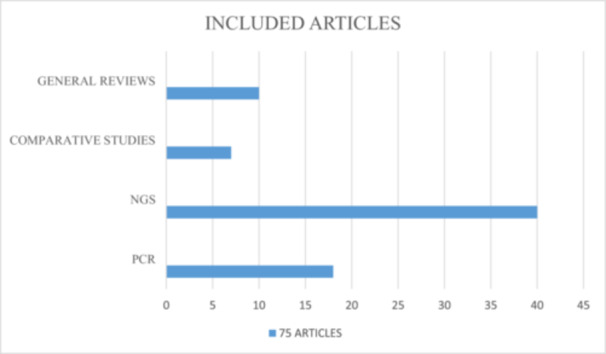
Each article included in the current scoping review was assigned in one of four categories: (1) Polymerase chain reaction (PCR)‐related studies: *n* = 18; (2) Next Generation Sequencing (NGS)‐related studies: *n* = 40; (3) comparative studies, including systematic reviews (SR) and meta‐analyses (MA), between different molecular diagnostic methodologies: *n* = 7; (4) general reviews on applying nucleic acid‐based strategies to detect PJIs: *n* = 10.

As Figure 1 (PRISMA flowchart) described, the study selection process included 75 articles: these articles were included after a preliminary review of 1315 records from multiple databases, including PubMed, Embase, Scopus, and Cochrane Library. Each article was included in one of four categories to satisfy the secondary purpose of this scoping review: (1) PCR‐related studies: *n* = 18; (2) NGS‐related studies: *n* = 40; (3) comparative studies, including SR and MA, between different molecular diagnostic methodologies: *n* = 7; and (4) general reviews on nucleic acid‐based strategies to detect PJIs: *n* = 10 (Figure 2).

#### PCR for microorganism identification

Results from two different PCR techniques have been included in the current review: end‐point PCR and quantitative PCR (qPCR). Unfortunately, end‐point PCR allows for only a qualitative (not quantitative) assessment of the micro‐organism, does not give information regarding antibiotic sensitivity, and does not represent a multiplex method. Because of these limitations, an increase in time and reagent use is usually required [44]. On the other side, qPCR allows for quantitative analysis and multiplexing, where the PCR target is amplified for multiple bacteria. Li et al. [34], comparing several PCR types (real‐time PCR, PCR sequencing, multiplex PCR, PCR lateral flow immunoassay) with different target genes (16S rRNA, 16S rDNA and specific primer) reported a synovial fluid pool sensitivity of 0.70 (95% confidence interval [CI], 0.66–0.74), a pooled specificity of 0.92 (95% CI, 0.90–0.93), a positive likelihood ratio (PLR) of 9.09 (95% CI, 5.28–15.67, a negative likelihood ratio (NLR) of 0.32 (95% CI, 0.24–0.42) and a diagnostic odds ratio (DOR) of 37.4 (95 CI, 17.77–78.74), respectively. In the same study [34], the authors compared the synovial fluid sensitivity and specificity in microorganism identification of several types of PCR to conventional fluid culture: similar sensitivities were observed (culture 70%; PCR 69%) while the specificity of synovial PCR was slightly lower than culture (culture 98%; PCR 91%).

Automated multiplex PCR (mPCR) has rapidly become an ideal, perioperative PJI diagnostic test. Many pharmaceutical companies have recently commercialised mPCR technologies for several settings, including SeptiFast by Roche (Basel, Switzerland), GeneXpert by Cephaid (Sunnyvale, CA, USA), Unyvero ITI G2 (Curetis, Holzgerlingen, Germany), Genotype by Hain Lifescience (Nehren, Germany) and BioFire JI (bioMérieux, Marcy l'Etoile, France). The BioFire JI panel has been recently included in two PJI diagnostic protocols which include the routinary use of mPCR [21, 56, 57]. Sangaletti et al. [57] proposed a stepwise approach to acute PJI while Ghirardelli et al. [21] showed that mPCR was the determinant test for microorganism identification in 63% of their PJIs. Recently, several authors [21, 57, 68] have recommended the intra‐operative use of mPCR (<60 min) in culture‐negative PJIs and doubtful revision cases to select the optimal surgical solution, including Debridement Antibiotic Implant Retention (DAIR), Debridement, Antibiotic Pearls, Retention of the Implant (DAPRI) [5], one‐stage, 1.5 stage and two‐stage revisions. Few authors evaluated the sensitivity and specificity of the BioFire JI panel in native and replaced joints: Esteban et al. [12] reported a sensitivity of 90.9% or greater for all but six organisms; Gaillard et al. [16], comparing the BioFire JI panel to standard culture reported a positive percentage agreement of 84.9% (95% CI, 78.8%–89.8%) and a negative percentage agreement of 100% (95% CI, 97.2%–100%); Pascual et al. [52] demonstrated overall high agreement between multiplex PCR and standard culture for both septic arthritis in native knees (88.4 %) and PJI (85.7 %); finally, Saeed et al. [55], in a comparative study between multiplex PCR and standard culture, showed an overall positive percent agreement of 91.6% and overall negative percent agreement of 93% for the BioFire JI panel compared to culture results.

Several authors also reported on the sensitivity and specificity of the BioFire JI panel in identifying the microorganism during revision surgery: Gardete‐Hartmann et al. [19] showed sensitivity and specificity of 41.4% (95% CI, 33.7–49.5) and 91.1% (95% CI 84.7–94.9), respectively; Moran et al. [46] showed a positive percent agreement (PPA) of 71.4% and a negative percent agreement (NPA) of 94.8% when fresh synovial fluid was analysed; finally, Berinson et al. [2] reported 100% sensitivity and specificity of the syndromic multiplex PCR to detect in‐panel microorganisms in bacterial arthritis. Many authors [2, 19, 46], including the current [21], highlighted the limited number of microorganisms present in the syndromic multiplex PCR as a major limitation of this kind of PCR technology.

Few authors reported on the diagnostic accuracy of the Unyvero ITI G2 system on synovial fluid alone: Jacobs et al. [29], in a consecutive series of unexpected PJI in aseptic knee revisions, showed a 96.8% specificity and a 96.8 % NPV of the mPCR compared to tissue cultures but were unable to report sensitivity and PPV; Lausmann et al. [33] reported a sensitivity of 85.1% and a specificity of 98.0%; Sigmund et al. [60] reported a sensitivity of 71.1% and a specificity of 96.2%; Suren et al. [63] reported, in a small series, a sensitivity of 80% and a specificity of 100%; Ludemann et al. [41] reported a surprising low sensitivity (33%) and a 91% specificity; finally, in a recent follow‐up study of 200 presumed aseptic hip and knee revision, van Schaik et al. [68] reported no additional value in predicting underlying PJI from the use of this mPCR system (Table 1).

**Table 1:** Review and comparison of available literature on mPCR systems applied to synovial fluid analysis for PJI detection in revision TKA.

Study	mPCR	Target	Pat.	PJI	SN	SP	PPV	NPV
Gardete‐Hartmann et al. [19]	BioFire	Hip/knee Revisions	195	141	41.4%	91.1%	NR	NR
Moran et al. [46]	BioFire	Native + Prosthetic	104	12	NR	NR	71.4%	94.8%
Berinson et al. [2]	BioFire				100%	100%		
Lausmann et al. [33]	Unyvero	Septic Aseptic Revisions	97	47	85.1%	98%	97.6%	87.5%
Sigmund et al. [60]	Unyvero	Septic Aseptic Revisions	90	38	71.1%	96.2%	93.1%	82.0%
Suren et al. [63]	Unyvero	Septic Aseptic Revisions	26	15	80%	100%	100%	77%
Jacobs et al. [29]	Unyvero	Presumed Aseptic Revisions	200	14	36.4% Hips 96.8% Knees	96.6% Hips 96.8% Knees	57.1% Hips	92.5% Hips
Lüdemann et al. [41]	Unyvero	Septic Aseptic Revisions	50	14	33%	91%	57%	NR

Abbreviations: mPCR, multiplex polymerase chain reaction; NPV, negative predictive value; NR, not reported; PJI, periprosthetic joint infections; PPV, positive predictive value; SN, sensitivity; SP, specificity; TKA, total knee arthroplasty.

#### NGS‐related studies

NGS represents an extremely powerful tool able to detect a single gene in 10 μL of synovial fluid throughout massive parallel sequencing of the nucleic acids present in a sample [28]. PCR‐generated amplicons are first separated and then monitored and sequenced in parallel. NGS sequencing includes nanopore sequencing, sequencing by synthesis, and by ligation: both amplicon targeted NGS (i.e., the bacterial 16S rDNA gene) and metagenomic NGS (mNGS) have been proposed as clinically relevant. Gu et al. [25] supported the use of mNGS over other molecular diagnostic techniques because of the possibility of identifying known and unexpected pathogens in an unbiased approach. Tan et al. [64], in a recent systematic review and meta‐analysis on the effectiveness of mNGS in PJI diagnosis, reported high sensitivity (93%) and specificity (95%) of mNGS in a selected series of 10 studies: unfortunately, only three studies (including 140 out of 955 patients) reported on exclusive synovial fluid analysis, which represents the optimal biologic material for PJI diagnosis in the strictly perioperative period. In a similar systematic review, Tang et al. [65] reviewed nine studies (five of them analysing preoperative and intraoperative synovial fluid samples only), reporting diagnostic sensitivities and specificities of NGS from 63% to 96% and 73% to 100%, respectively; in the same study, the detection rate of NGS for culture‐negative PJI patients in six studies was higher than 50% (range from 82% to 100%), while in three studies it was lower than 50% (range from 9% to 31%). In a recent retrospective, mNGS study on microorganism identification in PJI, Shi et al. [59] reported a sensitivity of 89.13%, a specificity of 94.74%, a positive predictive value of 97.62%, a negative predictive value of 78.26%, and an overall diagnostic accuracy of 90.77%; although mNGS was able to detect most of the pathogens identified by traditional microbial culture, four patients (8.6%) had false‐negative results for pathogens in this study. Fida and Tande [13] reviewed the published evidence of mNGS in PJI diagnosis in a “state of the art” paper, confirming a pooled diagnostic sensitivity and specificity of 93% and 95%, but also underlining major limitations, including the overwhelming presence of host DNA, contamination during processing, the need for specialised bioinformatics tools, and a lack of guidance on the interpretation of bioinformatics data. Li et al. [36], in an interesting prospective study, studied the clinical impact of mNGS in the decision‐making algorithm in a large cohort of patients with suspected PJI: in their study, the authors showed that only in 22.4%, synovial fluid mNGS was the determinant test to modify the clinical treatment. Because of this finding, the same authors (32) recommended utilising synovial fluid mNGS only in selected clinical scenarios, like previous PJIs, polymicrobial PJIs, and culture‐negative scenarios. Finally, Mei et al. [45], in a small cohort study, reported that mNGS had a high sensitivity (85.7%) but a moderate specificity (60%) and accuracy (65.2%) for the diagnosis of polymicrobial PJIs, which represents a worrisome scenario to many adult reconstruction surgeons.

Poulsen et al. [54] reported the diagnostic accuracy and value of 16S/18S targeted‐NGS (amplicon‐targeted) in the synovial fluid analysis of 87 samples: those authors reported a sensitivity and specificity of 44% and 100%, respectively; the positive predictive value and negative predictive value were 100% and 67% respectively. The median tNGS turnaround time in the same study [54] was 12 days, making this approach suboptimal for strictly perioperative use. 16S targeted‐NGS has also been evaluated by Goswami et al. [23] in culture‐negative PJI: the pathogen could be identified by NGS in 65.9% of culture‐negative patients; unfortunately, no turnaround time was presented in this study.

Many t‐NGS‐ and mNGS‐based studies have been evaluated by the current authors, but the data reported lacked power analysis, and they were not reported in the current section: Table 2 shows the selected literature on NGS systems applied to synovial fluid analysis for PJI detection.

**Table 2:** Review and comparison of available literature on NGS systems applied to synovial fluid analysis for PJI detection.

Study	NGS	Target	Samples from	Pat.	SN	SP	PPV	NPV
Tan et al. [64]	mNGS	PJI	Hip and Knee	155	93%	95%	18.3	0.07
Tang et al. [65]	NGS	PJI	Mix arthroplasties	1007	63%–96%	73%–100%	71%–100%	74%–95%
Shi et al. [59]	mNGS	PJI	Hip and Knee	46	89.13%	94.74%	97.62%	78.26%
Fida et al. [13]	mNGS	PJI	Mix	792	Up to 95%	Up to 95%	NR	NR
Li et al. [36]	mNGS	PJI	Hip and Knee	107	73.83%	91.49%	90.80	75.44%
Mei et al. [45]	mNGS	Polymicr. PJI	Hip and Knee	69	85.7%	60%	35.3%	94.3%
Poulsen et al. [54]	tNGS	PJI	Mix	87	44%	100%	100%	67%

Abbreviations: mNGS, metagenomic Next Generation Sequencing; NGS, Next Generation Sequencing type; NLR, negative likelihood ratio; NPV, negative predicting value; PJI, periprosthetic joint infection; PLR, positive likelihood ratio; PPV, positive predicting value; SN, sensitivity; SP, specificity.

#### Comparative studies, including SR and MA, between different molecular diagnostic methodologies

Seven studies were included in this results subsection. Six studies [18, 26, 28, 30, 32, 42] compared microbial detection rates between NGS/mNGS and standard culture while one [37] compared the accuracy of different NGS technologies. In their review, Indelli et al. [28] showed an NGS sensitivity of up to 94% and an NGS specificity of up to 100%; in the same study, the authors showed a culture sensitivity of up to 92% and a culture specificity of up to 100%. In their systematic review, Kato et al. [30] compared the detection rate of pathogens between NGS and microbial cultures utilising synovial fluid samples aspirated from replaced joints: the detection rate in NGS was significantly higher than in culture (OR 4.52, 95% CI 2.86–7.16). Different findings were shown by Kildow et al. [32], the authors analysed samples from 116 patients with a suspect of PJI. They showed that NGS did not provide superior sensitivity or specificity results compared to culture. Gao et al. [18], in a small case series of synovial fluid samples from patients previously treated with antibiotics, compared the sensitivity of mNGS and standard culture: mNGS showed a higher sensitivity (68.1% vs. 25.5%, *p* < 0.01). Hantouly et al. [26], in a systematic review and meta‐analysis, compared the sensitivity and accuracy of NGS and standard culture: data from 341 patients (seven studies) were reviewed and the overall pooled sensitivity of NGS was 94% while the pooled specificity was 89%; on the other side, the pooled sensitivity of culture was 70% while the specificity was 94%. In the same study [26], the accuracy of NGS and culture were 91.9% and 80.5%, respectively. Mahnic et al. [42], in a recent study, compared 16S amplicon metagenomic sequencing with traditional culture techniques: the sensitivity of 16S AS compared to cultivation was 27/33 (81%). Finally, Li et al. [37], comparing different sequencing arrays in a systematic review and meta‐analysis of 12 studies with 1965 patients included, showed that sequencing by synthesis, a time‐saving and lower‐cost NGS method, seemed to have better specificity than other NGS methods but had a similar specificity when compared with Sanger sequencing.

#### General reviews on the application of nucleic acid‐based strategies to detect PJIs

Ten studies were included in this section. Oliva et al. [50] and McClure et al. [44] reviewed the results of the application of multiple diagnostic techniques to detect microorganisms in orthopaedic implant‐related infections. Oliva et al. [50] recommended using molecular and metabolic assays as complementary to culture‐based methods to shorten the time for pathogen identification, to search for fastidious microorganisms or when antibiotics have been previously administered: in particular, the new generation of multiplex PCR, providing results within 1 h against several days for synovial fluid culture, offered the option of faster microorganism identification and targeted antimicrobial therapy in multiple PJI scenarios. McClure et al. [44], comparing different acid‐based diagnostic strategies, also recommended using quantitative PCR (qPCR) methods when microorganism identification represented the determinant factor in the treatment decision‐making process: in fact, qPCR is adaptable for rapid point‐of‐care testing even in the operating room while the time required for NGS testing (from data analysis to interpretation) is currently impractically long. Portillo and Sancho [53], in a review that compared non‐culture techniques based on nucleic acid amplification and sequencing methods, recommended caution before considering NGS diagnostic methods as the standard of care, since the interpretation of results must be based on strict criteria and an understanding of bioinformatics to rule out contaminants and avoid overrepresentation of host DNA, which may ultimately interfere with the microorganisms' DNA sequences. Davis et al. [8], in a recent review, underlined multiple advantages of synovial fluid PCR over other diagnostic tools, including the possibility of detecting pathogens not growing under traditional culture conditions, not being affected by the recent use of antibiotics, and its potential for faster turnaround times than culture‐based methods. Esteban Gomez‐Barrena [11], in a recent review supported by the European Federation of National Associations of Orthopaedics and Traumatology (EFORT) society, presented an update about molecular biology techniques to detect orthopaedic implant‐related infections to give practical recommendations to support the decision‐making process in PJI scenarios. Those authors [11] suggested reserving PCR‐based molecular biology methods for patients with negative cultures (up to 42% in their series) and synovial fluid samples with doubtful significance. The same authors [11] considered the new metagenomic methodologies experimental since they require understanding the relevance of all the microorganisms detected in a single sample. Gamie et al. [17], in an expert review of molecular sequencing technologies in PJI management, underlined that PCR, especially by synthesis and multiplex PCR, complemented culture and showed good specificity and positive predictive value. The same authors [17] showed more enthusiasm towards NGS and mNGS techniques with respect to other authors [32, 50, 53], highlighting that mNGS has demonstrated accuracy that inspires cautious optimism both in culture‐positive and culture‐negative PJIs. Gatti et al. [20], in a review focused on the application of multiple diagnostic techniques to guide the surgical intervention in PJI, highly recommended implementing molecular biology techniques which showed the capability of reducing dramatically the turn‐around time to a few hours and completely automated the workflow: between them, synovial fluid PCR showed the ability to solve the culture‐negative PJI dilemma thanks to its increased sensitivity; nevertheless, the main limitation of PCR‐related techniques remains the use of specific primers depending on the investigated organism. Zhou et al. [70], in a review aiming to relate PJI surgical management strategies to the accuracy of the diagnostic technology, confirmed that the success rate of any implant retaining procedure (i.e., DAIR) increased when the infecting microorganism was identified through molecular diagnostics. Mazzella et al. [43] expressed caution on the wide use of NGS as a determinant test for microorganism identification as guidance in the PJI decision‐making process: to those authors' knowledge, NGS has not been cleared by the Food and Drug Administration (FDA) for PJI diagnosis, although it can be available as a Laboratory Developed Test (LDT) with lab‐dependent panels of genes, recommending the use of NGS techniques mainly in culture‐negative samples. Finally, Wouthuyzen‐Bakker [69] presented an innovative, molecular techniques‐based, diagnostic flow chart for culture‐negative PJI: multiplex PCR was recommended as a first‐line test in postoperative, acute PJIs, and late chronic PJIs with a sinus tract; the use of 16S PCR was recommended in all cases of chronic PJIs without the presence of a sinus tract. In the same study, those authors [69] recommended using NGS only as an alternative diagnostic method to identify atypical microorganisms, especially in acute PJI scenarios.

## DISCUSSION

This scoping review aimed to provide clinical recommendations regarding the role of molecular diagnostics in decision‐making for PJI treatment. The most significant finding was that molecular diagnostic methods hold substantial perioperative value in identifying the causative microorganisms in both acute and chronic PJIs.

mPCR demonstrated up to 85% sensitivity and 100% specificity [2, 19, 46] in the identification of microorganisms in acute PJI: multiple authors recommended mPCR as the optimal technology for intraoperative use (<60 min) in all acute, culture‐negative PJIs [44, 69]. The authors from the current study group adhere to the 2018 International Consensus Meeting definition [6] of acute PJI: a PJI is considered acute when patients present symptoms for 4 weeks or less, regardless of the PJI etiopathogenesis (acute postoperative vs. hematogenous). Microorganism identification in acute PJI has been shown to increase the success rate of implant‐saving procedures compared to culture‐negative acute PJI [14, 27]. High‐virulent microorganisms have classically caused acute PJIs: while *Staphylococcus aureus, Streptococci* spp., *Enterococcus*, and other Gram‐negative bacilli *have* prevailed in many series focusing on an intraoperative origin, *Streptococcus pneumoniae, Escherichia coli, Klebsiella*, and *Enterobacter* have been frequently reported as responsible microorganisms for hematogenous spreading [20]. For this reason, mPCR (i.e., Biofire JI Panel, bioMérieux, France), which allows for the amplification of multiple bacteria in the PCR target, represents an ideal tool for perioperative pathogen identification; however, multiplex PCR systems sometimes lack accuracy in identifying low‐virulent bacteria [41, 58, 68]. Another PCR technique, 16S PCR, has been proposed in several articles reviewed by the current authors: unlike mPCR, 16S PCR has the significant limitation of detecting only the most abundant microorganism in the synovial fluid, potentially missing polymicrobial PJIs; [34] due to this limitation, a few authors have classified 16S PCR as a second‐line molecular diagnostic test [69].

Multiple authors have recommended NGS for identifying microorganisms in all culture‐negative and PCR‐negative acute PJIs [17, 30, 44, 64, 69]. Early postoperative PJIs are often described as polymicrobial [15, 40], requiring NGS or mNGS to sequence the entire microbial genome obtained during synovial fluid analysis. The advantage of using mNGS in culture‐negative and PCR‐negative PJIs relates to its capability for unbiased sampling. In their review, Goswami and Parvizi [24] demonstrated that NGS technologies could identify two‐thirds of culture‐negative cases. However, NGS and mNGS present major drawbacks in microorganism identification for both acute and chronic PJIs, including the burden of sample preparation, analysis of large data sets, equipment costs, high operating expenses, difficulties in detecting pathogen virulence, and contamination from background microorganisms [17, 64].

Nevertheless, the preliminary identification of the PJI causative microorganism, thanks to molecular diagnostics and targeted local delivery of antibiotics, decreased the postoperative length of the antibiotic treatment [9, 39] in acute and chronic PJI.

Many reports [8, 11, 20, 43, 44, 50, 69] evaluated in the current scoping review recommended the use of molecular diagnostics also in chronic PJI scenarios, typically characterised by a culture negativity rate as high as 50% [22]. From a surgical treatment standpoint, in chronic PJI, identifying the causative microorganism plays a major role: Kildow et al. [31] included microorganism identification as mandatory to proceed with a single‐stage revision for PJI. Historically, low‐virulent pathogens such as coagulase‐negative *Staphylococci* (CoNS), especially *Staphylococcus epidermidis* and *Cutibacterium* spp., have been considered causative organisms for chronic PJI: the identification of these pathogens through standard culture has been classically more difficult because of the lack of an appropriate medium, a short incubation time and prior antibiotic therapy [61]. Synovial fluid PCR showed better accuracy than culture in identifying PJIs caused by low‐virulence bacteria [47], especially in patients who received antibiotics within 30 days before synovial fluid analysis [34]. NGS and mNGS showed accuracy in identifying low‐virulence bacteria, typically causing chronic PJIs, in many studies evaluated in the current scoping review [59, 64, 70], especially when the aetiology was polymicrobial [45]. Unfortunately, like in acute PJIs, NGS techniques have not been recommended as a first line, 'alone tool' for microorganism identification in chronic PJIs due to the high risk of false positives [45].

The secondary purpose of this scoping review was to present recommendations for the surgical management of PJI with multiple surgical approaches available, including DAIR/DAPRI, single‐stage, 1.5‐stage, and two‐stage revision arthroplasty, among others. Multiple authors considered the preoperative or intraoperative identification of the PJI causative organism and the timeframe between symptoms appearance and treatment (acute vs. chronic PJI) as the key factors for the success of the surgical approach [31, 62]. The current scoping review confirmed that the microorganism detection time varied between standard culture and molecular diagnostics. Multiple authors showed that the turnaround times (TAT) for standard culture averaged 3 days for bacteria, 7 days for fungi, and 45 days for mycobacteria [17, 30, 45]. Multiplex PCR detected the causative microorganism in 1 h [21], while the typical NGS and mNGS TAT was reduced to less than 48 h [44].

**Figure 3:**
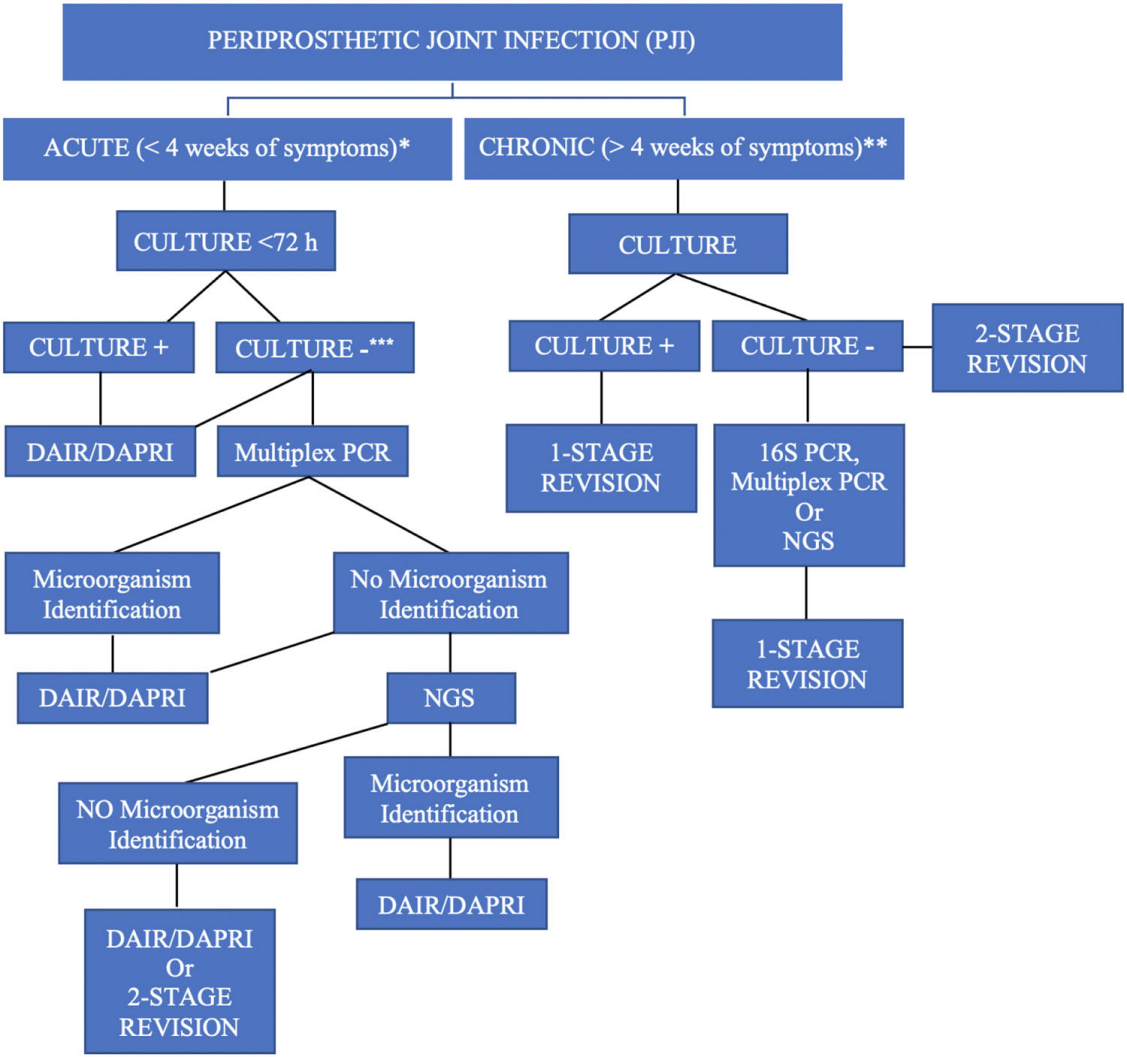
Diagnssostic‐treatment flow chart for acute and chronic periprosthetic joint infections (PJI). The definition of a PJI as acute or chronic has been formulated according to the 2018 International Consensus Meeting on PJI as reported by Chotanaphuti et al. [6]. ACUTE*: Contraindications to DAIR include the presence of sinus tract, DTT infections, loosening of the implant, and antibiotic availability [3, 7]. There is still a lack of consensus on whether to perform DAIR without identifying micro‐organisms: a recent systematic review does not deem pathogen identification necessary before carrying out a DAIR procedure in an acute PJI setting [38]. CULTURE‐NEGATIVE***: In the presence of a sinus tract, the use of molecular diagnostics needs to be supported by future research [58]. CHRONIC INFECTIONS**: Contraindications to a 1‐stage revision also include the presence of sepsis, an infection caused by drug‐resistant bacteria or DTT, the presence of a sinus tract, severe soft‐tissue deficiency over the joint and no history of multiple revisions [35, 51, 58]. The investigation for the infecting pathogen should include a blood culture (if the patient is suspected of having a hematogenous PJI), a urinalysis (if the patient is suspected of having a urinary tract infection), evaluation for distant focus according to the pathogen and clinical signs, and evaluation for a contiguous infection. Few authors have supported chronic, suppressive antibiotic therapy as an alternative to surgical intervention in selected clinical scenarios [4]. DAIR: Debridement, Antibiotics, Implant Retention; DAPRI: Debridement, Antibiotic Pearls Retention of the Implant [5]. DTT: difficult‐to‐treat infections caused by pathogens resistant to biofilm‐active antimicrobials [7].

Following the aforementioned considerations on the sensitivity, specificity, and possible applications of molecular diagnostics in PJI scenarios, the current authors present a stepwise diagnostic algorithm useful for patients in the clinical setting (Figure 3). The flow chart presented here takes into consideration the clinical utility and intrinsic limitations of several molecular diagnostics reviewed by the current authors. If end‐point PCR and qPCR are widely adapted in the clinical setting, including for rapid point‐of‐care testing in the operating room, amplicon‐targeted NGS and mNGS are currently used primarily for research [44].

Several limitations of this scoping review need to be emphasised. First, the analysed literature showed that culture‐independent diagnostic tools exist, and many of them have been applied to clinical practice; unfortunately, many molecular diagnostic technologies are not ready for general use due to economical, technical, and logistical obstacles involving the lab infrastructure needed for any type of nucleic acid‐based analysis. Second, this scoping review was designed to assist orthopaedic surgeons in the PJI treatment decision‐making process, focusing on the strict perioperative period. In those scenarios, the turnaround time plays a significant role, and few of the newer molecular diagnostic technologies (i.e., NGS) still require a diagnostic timeframe similar to standard culture, limiting their use. Third, our scoping review focused on studies reporting synovial fluid analysis data only: data on tissue biopsies and sonicated fluid were reviewed but ultimately not included in the current discussion. In this instance, two diagnostic technologies appeared very promising to the current authors: Noone et al. [49] presented a rapid (<24 h) and accurate, nanopore‐based mNGS diagnostic protocol on tissue biopsies; Echeverria et al. [10] showed that blood microbial cell‐free DNA sequencing (cfDNA) holds promise in improving surgical and pharmacological care of PJI by increasing the number of cases in which pathogens are identified, enabling higher confidence and more rapid species identification, and by potentially providing a test to monitor the efficacy of pathogen clearance by the chosen treatment strategy. Finally, the diagnostic flow chart presented here was intended for general use, knowing that the availability of molecular diagnostics varies significantly worldwide.

## CONCLUSION

The current scoping review confirmed that, in modern arthroplasty, molecular diagnostics are becoming extremely valuable tools in the decision‐making process of the surgical treatment of periprosthetic joint infections. To date, culture‐based techniques still represent the gold standard in PJI microorganism identification, but our review showed that standard culture, in 2025, could be integrated with newer nucleic acid‐based strategies. However, many molecular diagnostics (especially NGS‐ and multiplex PCR‐based) techniques still present difficulties interpreting the sequencing results. Large‐size RCT studies are needed to confirm the clinical value of molecular diagnostics in periprosthetic joint infection patients.

## AUTHOR CONTRIBUTIONS

All authors contributed to the conception and design of the study. Pier Francesco Indelli, Chiara Maci, Antonella Castagna, Laura Schianchi, Chiara Tassan Din, James Harty, Goksel Dikmen, Christian Schaller, and Marko Ostojić participated in data collection. Pier Francesco Indelli, Trifon Totlis, Bruno Lovreković, Michael Engl, Bruno Violante, Pawel Skowronek, Guillaume Demey, and Stefano Ghirardelli revised the manuscript. All authors reviewed and approved the final version of the manuscript.

## CONFLICT OF INTEREST STATEMENT

Pier Francesco Indelli, MD, PhD, is a consultant for medical education for bioMérieux (France) and received research support from Biocomposites (UK). The other authors declare no conﬂicts of interest regarding the manuscript's content.

## ETHICS STATEMENT

This study was conducted in accordance with the principles of the Declaration of Helsinki. As a scoping review of previously published articles, this study did not require approval from the Internal Review Board (IRB).

## Supporting information

Search Terms Supporting material.

## Data Availability

The data sets used and/or analysed during this study are available from the corresponding author upon reasonable request.
