# Supplementary material for: Molecular diagnostics for perioperative microbial identification in periprosthetic joint infection: A scoping review and proposal of a diagnostic flow chart
Source: J Exp Orthop. 2025 May 5;12(2):e70263. doi: 10.1002/jeo2.70263 (PMC12051374; doi:10.1002/jeo2.70263)
Supplement: Supplementary file 1 — Search Terms Supporting material. [file JEO2-12-e70263-s001.docx]

The search terms included:

Population: ((((((((((((((((((((((((((((((((Arthroplasty, Replacement, Knee[MeSH Terms]) OR (Arthroplasties, Replacement, Knee)) OR (Arthroplasty, Knee Replacement)) OR (Knee Replacement Arthroplasties)) OR (Knee Replacement Arthroplasty)) OR (Replacement Arthroplasties, Knee)) OR (Replacement Arthroplasty, Knee)) OR (Replacement, Total Knee)) OR (Total Knee Replacement)) OR (Knee Replacement, Total)) OR (Knee Arthroplasty)) OR (Arthroplasty, Knee)) OR (Arthroplasties, Knee Replacement)) OR (Knee Arthroplasty, Total)) OR (Arthroplasty, Total Knee)) OR (Total Knee Arthroplasty)) OR (Arthroplasty, Replacement, Partial Knee)) OR (Unicompartmental Knee Arthroplasty)) OR (Arthroplasty, Unicompartmental Knee)) OR (Knee Arthroplasty, Unicompartmental)) OR (Unicompartmental Knee Replacement)) OR (Knee Replacement, Unicompartmental)) OR (Partial Knee Replacement)) OR (Knee Replacement, Partial)) OR (Unicondylar Knee Replacement)) OR (Knee Replacement, Unicondylar)) OR (Unicondylar Knee Arthroplasty)) OR (Arthroplasty, Unicondylar Knee)) OR (Knee Arthroplasty, Unicondylar)) OR (Partial Knee Arthroplasty)) OR (Arthroplasty, Partial Knee)) OR (Knee Arthroplasty, Partial)) OR ((((((((((((((((((((Arthroplasty, Replacement, Hip[MeSH Terms]) OR (Hip Replacement Arthroplasty)) OR (Replacement Arthroplasties, Hip)) OR (Replacement Arthroplasty, Hip)) OR (Hip Prosthesis Implantation)) OR (Hip Prosthesis Implantations)) OR (Implantation, Hip Prosthesis)) OR (Prosthesis Implantation, Hip)) OR (Arthroplasties, Hip Replacement)) OR (Arthroplasties, Replacement, Hip)) OR (Hip Replacement Arthroplasties)) OR (Arthroplasty, Hip Replacement)) OR (Hip Replacement, Total)) OR (Total Hip Arthroplasty)) OR (Arthroplasty, Total Hip)) OR (Hip Arthroplasty, Total)) OR (Total Hip Arthroplasties)) OR (Replacement, Total Hip)) OR (Total Hip Replacements)) OR (Total Hip Replacement)) AND ((((((((((Prosthesis-Related Infections[MeSH Terms]) OR (Prosthesis Related Infections)) OR (Infections, Prosthesis-Related)) OR (Prosthesis Related Infection)) OR (Infection, Prosthesis Related)) OR (Related Infection, Prosthesis)) OR (Related Infections, Prosthesis)) OR (Prosthesis-Related Infection)) OR (Periprosthetic joint infection*)) OR (PJI)) OR (Septic arthritis).

Concept: (((((((((((((((((((((Molecular Diagnostic Techniques[MeSH Terms]) OR (Diagnostic Technique, Molecular)) OR (Diagnostic Techniques, Molecular)) OR (Molecular Diagnostic Technique)) OR (Technique, Molecular Diagnostic)) OR (Techniques, Molecular Diagnostic)) OR (Molecular Testing)) OR (Testing, Molecular)) OR (Molecular Diagnostic Technics)) OR (Diagnostic Technic, Molecular)) OR (Diagnostic Technics, Molecular)) OR (Molecular Diagnostic Technic)) OR (Technic, Molecular Diagnostic)) OR (Technics, Molecular Diagnostic)) OR (Molecular Diagnostic Testing)) OR (Diagnostic Testing, Molecular)) OR (Testing, Molecular Diagnostic)) OR (Molecular diagnostic technology*)) OR (Pathogen detection)) OR (multiplex PCR)) OR (Next generation sequencing)) OR (NGS).
